# The influence of colour and sound on neuronal activation during visual object naming

**DOI:** 10.1016/j.brainres.2008.08.037

**Published:** 2008-11-19

**Authors:** Julia Hocking, Cathy J. Price

**Affiliations:** a Centre for Magnetic Resonance, The University of Queensland, Brisbane, Australia; b Wellcome Trust Centre for Neuroimaging, UCL, London, UK

**Keywords:** Object naming, Perceptual, Conceptual, Integration, Audiovisual, Crossmodal

## Abstract

This paper investigates how neuronal activation for naming photographs of objects is influenced by the addition of appropriate colour or sound. Behaviourally, both colour and sound are known to facilitate object recognition from visual form. However, previous functional imaging studies have shown inconsistent effects. For example, the addition of appropriate colour has been shown to reduce antero-medial temporal activation whereas the addition of sound has been shown to increase posterior superior temporal activation. Here we compared the effect of adding colour or sound cues in the same experiment. We found that the addition of either the appropriate colour or sound increased activation for naming photographs of objects in bilateral occipital regions and the right anterior fusiform. Moreover, the addition of colour reduced left antero-medial temporal activation but this effect was not observed for the addition of object sound. We propose that activation in bilateral occipital and right fusiform areas precedes the integration of visual form with either its colour or associated sound. In contrast, left antero-medial temporal activation is reduced because object recognition is facilitated after colour and form have been integrated.

## 1 Introduction

It is well documented that visual object processing proceeds in a hierarchy of stages with early sensory input activating primary visual occipital cortices and later stages of visual processing progressing anteriorly along ventral aspects of the occipito-temporal lobes (for a review see Grill-Spector, 2003). Although a similar hierarchical model to visual processing has been proposed for auditory objects (Clarke et al., 2000; Maeder et al., 2001; Rauschecker and Tian, 2000), it is not entirely clear how multimodal perceptual inputs are integrated into an amodal conceptual representation. In this paper we investigate how visual object naming is influenced by the addition of appropriate colour or sound.

Behaviourally, object naming is facilitated when congruent perceptual cues are increased. This has been shown within the visual modality when objects are appropriately coloured (Humphrey et al., 1994; Mapelli and Behrmann, 1997; Ostergaard and Davidoff, 1985; Price and Humphreys, 1989) and across modality for audiovisual speech processing (Hershenson, 1962; Summerfield, 1992) and audiovisual nonverbal processing (Giard and Perronet, 1999; Laurienti et al., 2003; Teder-Sälejärvi et al., 2005). Additional perceptual cues are particularly advantageous when an object has multiple structurally similar neighbours. For example, 4-legged animals or round fruits have many structurally and semantically similar neighbours that compete for selection (Humphreys et al., 1995; Joseph and Gathers, 2003; Joseph and Proffitt, 1996; McRae et al., 1999). By increasing perceptual cues, for instance the provision of appropriate colour, competition is reduced and response times are facilitated.

Functional neuroimaging studies have associated the perceptual facilitation with differential neuronal responses. For example, Moore and Price (1999) reported a relative decrease in activation in bilateral anterior temporal cortices and a right posterior middle temporal region for appropriately coloured pictures of objects compared to black and white pictures of the same stimuli. In contrast combined auditory and visual information has been shown to increase activation in a network of regions, including the posterior superior temporal sulcus (pSTS; e.g. Beauchamp et al., 2004; Hein et al., 2007) and the antero-medial temporal lobe (e.g. Taylor et al., 2006).

The current literature therefore suggests that as the number and type of perceptual cues are increased, neuronal activation 1) increases in response to the presentation of crossmodal relative to uni-modal inputs, and 2) decreases when additional perceptual cues (e.g. form and colour) are presented within a modality. These contrasting patterns of activation could either be due to task differences (passive viewing versus naming) or the level of processing (perceptual or semantic) at which multimodal inputs are integrated within versus across modality. At the perceptual level, it has already been established that sensory processing in one modality can influence activation in another modality, for example, responses in auditory cortices to visual stimuli (Nyberg et al., 2000; Wheeler et al., 2000), and responses in visual cortices to auditory stimuli (Bookheimer et al., 1998). At the semantic processing level, functional imaging has shown that both visual and auditory inputs access a shared semantic system (Bookheimer et al., 1998; Booth et al., 2002; Spitsyna et al., 2006; Thierry and Price, 2006).

**Fig. 1:**
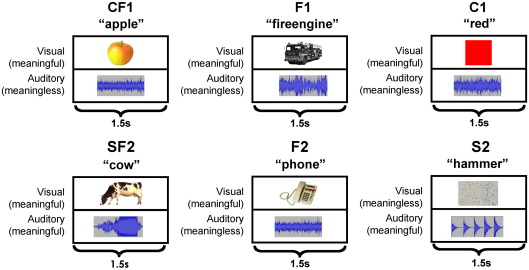
Example of a single stimulus trial for each of the six conditions. Visual stimuli were pictures of objects, colour patches or scrambled images. Auditory stimuli were object sounds or scrambled sounds, depicted here as the 1.5 s auditory sound envelope. Key: C = colour; F = form; S = sound; 1 = part of form and colour conditions; 2 = part of form and sound conditions.

The functional imaging study reported here investigates the influence of both colour and sound on object naming in the same group of participants. Colour–form integration was investigated by comparing appropriately coloured objects to their black and white counterparts and to coloured squares. Likewise, sound–form integration was investigated by comparing photographs of objects with their corresponding sounds to photographs only and sounds only. This experimental design (see Fig. 1 for details) enables the identification of regions where object naming activation is modulated by increased perceptual cues (1) independent of whether the additional perceptual cue is within or between modality (e.g. form and colour versus form and sound); and (2) dependent on whether the additional perceptual cue is within or between modality. Moreover, we can distinguish between the different levels at which colour or sound may influence neuronal patterns of activation. For example, if activation for two inputs (i.e. [form with colour] or [form with sound]) is higher than the average of each input alone (i.e. [form only plus colour only] or [form only plus sound only]), then the inputs must be influencing one another prior to or during the integration process. In contrast, if activation for two inputs is less than the average of each input alone, then the effect must be occurring after integration when two inputs have become one object. In addition to a whole brain analysis, previous studies provide us with a priori functional–anatomical hypotheses in two regions of interest. First, we expected to see decreased activation for colour–form integration in the bilateral medial anterior temporal and right posterior middle temporal regions reported by Moore and Price (1999). Second, we expected increased activation for sound–form integration in the left posterior superior temporal cortex (Beauchamp et al., 2004).

## 2 Results

### 2.1 Behavioural results

Means and standard deviations are shown in Table 1. For the colour-form integration, pairwise *t*-tests between conditions revealed that response latencies for object naming were facilitated with the presence of colour (*t* = 2.519, *p* < 0.015) and also significantly faster when naming colour patches relative to naming both black and white pictures (*t* = 8.801, *p* < 0.0005) and colour pictures (*t* = 6.122, *p* < 0.0005). For audiovisual integration, object naming latencies were neither facilitated nor inhibited when both sound and form information were simultaneously presented (*t* = 0.044, *p* = 0.965) although naming objects from their sound only was significantly slower than either form only (*t* = 13.062, *p* < 0.0005) or form with sound (*t* = 15.10, *p* < 0.0005). This effect for auditory naming is consistent with previous studies and may be due to the presentation duration of auditory relative to visual stimuli. Auditory recognition may not occur until stimulus presentation (up to 1500 ms) is complete, whereas picture presentation is complete at stimulus onset.

### 2.2 Functional imaging results

#### 2.2.1 Whole brain analysis

**Fig. 2:**
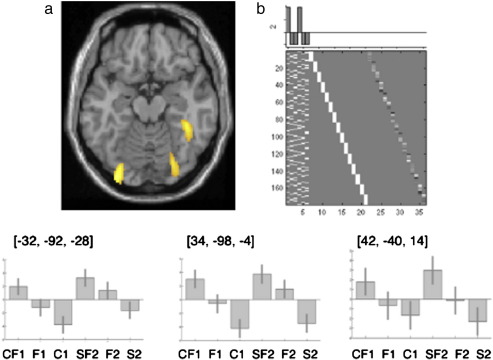
Main effect of increased perceptual input. (a) Activation increases for the addition of either colour or sound, rendered at *p* < 0.001 uncorrected on an averaged T1 axial slice of the standardized brain at *Z* = − 16. (b) Design matrix showing contrast weights [2− 1− 1 2− 1− 1]. Plots show mean-centred relative effect sizes at the peak co-ordinates of significant activations in left and right occipital and right anterior fusiform (see also Table 2). For Key see Fig. 1.

**Fig. 3:**
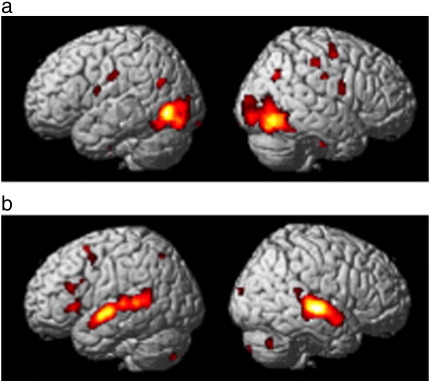
Main effect of visual versus auditory input. Activation increases for conditions involving (a) visual only > auditory only inputs i.e. contrast weights of [1 1 1 0 1− 4] and (b) auditory only > visual only inputs i.e. contrast weights of [− 1− 1− 1 0− 1 4]. Activation rendered at *p* < 0.001 uncorrected on the SPM standard surface model of an averaged brain, with a minimum cluster size of 10 voxels.

The main effect of additional perceptual cues (within and across modalities) was identified by comparing activation for the [form with colour; CF1] plus [form with sound; SF2] conditions to the corresponding [form only; F1, F2], [colour only; C1] and [sound only; S2] conditions. Activation was identified in bilateral occipital lobes and the right anterior fusiform (see Fig. 2 and Table 2). There was no significant decreased activation and no significant difference between the addition of colour or the addition of sound (relative to their corresponding components). As expected, the main effect of visual versus auditory inputs yielded activation predominantly in visual and auditory regions respectively (see Fig. 3).

#### 2.2.2 Regions of interest

**Fig. 4:**
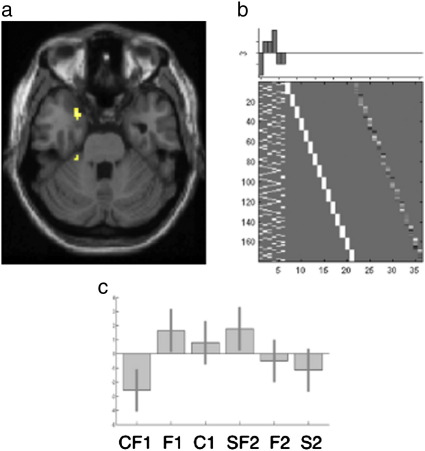
Interaction in antero-medial temporal lobe. (a) Activation in antero-medial temporal ROI, rendered on an averaged T1 section at *Z* = − 28. (b) Design matrix showing contrast weights [− 2 1 1 2− 1− 1] (c) Plot of mean-centred relative effect sizes for all conditions showing least activation for the combination of form and colour [− 22, 8,− 28]. For Key see Fig. 2. In addition the plot suggests a non-significant trend for higher activation in F1 then F2. This may be due to category differences because the natural stimuli in F1 were pictures of fruit and vegetables whereas the natural stimuli in F2 were pictures of animals (see Appendix for details). Critically however, all reported comparisons are within study and within category, therefore the difference does not alter the conclusions drawn.

For the [form with colour; FC1] condition relative to [form; F1] only and [colour; C1] only conditions, we expected decreased activation in bilateral antero-medial temporal cortex centred around [− 26, 0,− 20] and [30, 8,− 24] as well as in the right posterior temporal cortex [64,− 56, 0] as reported by Moore and Price (1999). This prediction was confirmed in the left medial anterior temporal region at [− 24, 8,− 30]; *Z* = 3.4; *p* = 0.023 after small volume correction, see Fig. 4. There was no corresponding effect in the right hemisphere regions or in any of the regions for the [form with sound; SF2] relative to [form only; F2] and [sound only; S2] conditions. Moreover, the difference in the effect of adding colour versus sound was confirmed by a significant interaction in the left medial anterior temporal region [− 22, 8,− 28, *Z* = 3.6; *p* < 0.05 following small volume correction].

For the [form with sound; SF2] condition relative to [form; F2] only and [sound; S2] only conditions, we expected increased activation in the left posterior superior temporal cortex centred around [+/− 50,− 56, 4 in MNI space] from [− 50,− 55, 7] in Talairach and Tournoux space as reported by Beauchamp et al. (2004). This effect was not significant in our data (*p* > 0.05, uncorrected in a sphere of 10 mm radius centred on [+/− 50,− 56, 4]).

## 3 Discussion

This experiment was designed to investigate the influence of increased perceptual information (colour or sound) on naming objects from their visual form. The results highlight two key findings. Firstly, independent of whether increased perceptual information was provided by uni-modal visual stimuli that were appropriately coloured, or by the combination of crossmodal audiovisual perceptual inputs, an enhanced response was observed in bilateral occipital cortices and the right anterior fusiform gyrus. The bilateral occipital regions are well established uni-modal visual association areas and, as such, correspond to areas involved in visual perception. Examination of the effect sizes across all conditions suggests that, like the bilateral occipital regions, the right anterior fusiform region is also driven by visual form processing. Specifically, activation was higher for form only than either sound only or colour only and there was no significant difference between sound only or colour only as one might expect if the area was involved in semantic processing. Nevertheless, the influence that colour and sound had on form processing indicates that these regions are also influenced by other perceptual inputs. This may explain why previous studies have associated the right anterior fusiform with amodal processing (Martin, 2007; Thierry and Price, 2006; Vuilleumier et al., 2002).

Secondly, the addition of colour but not the addition of sound reduced activation in a left antero-medial temporal region, as previously reported by Moore and Price (1999). This region has been strongly linked to semantic processing by neuropsychological studies of patients with semantic dementia and herpes simplex virus encephalitis (Barbarotto et al., 1996; Brambati et al., 2006; Davies et al., 2004; Kapur et al., 1994; Noppeney et al., 2007). Our data therefore suggest that the addition of colour decreased the demands on semantic processes in the left antero-medial temporal cortex, consistent with the faster naming times that we observed for the [form with colour] condition compared to form only condition (see Table 1). This apparent “facilitation” contrasts to the effect of additional colour in posterior cortices where activation increased in areas associated with both visual perception and amodal semantics. We now discuss each of these regional effects in turn.

### 3.1 The effect of colour and sound in posterior occipital regions

The posterior occipital activations associated with additional perceptual cues (colour or sound) are in well established visual processing areas (see Grill-Spector, 2003 for a review). Indeed, as shown in Table 2, bilateral occipital activation was most significant when object form was compared to no object form (colour only or sound only). Nevertheless, our results show that activation in these visual processing regions is modulated by the addition of both colour and sound relative to the individual components of form, colour or sound alone. The effect of combining form and colour on occipital activation is predicted on the basis of previous studies (Moore and Price, 1999; Zeki and Marini, 1998). Critically, however, the occipital areas we observe, after colour naming has been subtracted out, are at least 20 mm posterior to the areas associated with colour processing only (Bartels and Zeki, 2000; Dojat et al., 2006; Hadjikhani et al., 1998; Howard et al., 1998; McKeefry and Zeki, 1997; Nunn et al., 2002; Sakai et al., 1995; Zeki et al., 1991). This suggests that increased posterior occipital activation for the combined [form and colour] condition occurs at an early visual processing stage. Indeed, it is known that the extraction of information about an object's colour occurs early in visual analysis (Cant and Goodale, 2007) and that binding of visual attributes is subsequent to stimulus processing itself (Bartels and Zeki, 2006).

Increased posterior occipital activation for the combined form and sound condition is more surprising. However, other studies have also demonstrated modulation of visual areas by auditory inputs. For instance, Bookheimer et al. (1998) directly compared the semantic network involved in uni-modal auditory and uni-modal visual naming. Employing the same design as a visual object processing study (Bookheimer et al., 1995), subjects were scanned during an auditory semantic task whilst blindfolded, thus ensuring no external visual stimulation. In addition to brain regions typically associated with language processing (bilateral superior temporal, left inferior frontal, including Broca's and Wernicke's areas), activation was also observed in bilateral primary visual cortex, consistent with their previous study on visual object processing. They interpreted this result in terms of the influence from top–down semantic to sensory processing which may facilitate activation of semantic representations through an automatic evocation of visual images consequent to auditory processing. Despite these previous findings, the increased occipital activation that we observe in our study for combined auditory and visual inputs remains surprising because there is no need to evoke visual imagery when the relevant visual inputs are already present. Our study therefore suggests that auditory object processing automatically activates visual areas irrespective of whether visual processing is required or not.

In our behavioural study, there was no evidence that naming times were facilitated by the combination of form and sound. Moreover, facilitation is usually associated with reduced rather than increased activation. These observations lead us to propose that the effect of combined form and sound information in the posterior occipital regions reflects increased perceptual processing rather than processing related to an integrated percept. A possible explanation for this effect is that the additional perceptual information increased attention to the visual input thereby enhancing visual form processing. However, there are two points to note here. First the task was kept constant across all conditions to ensure that subjects attended to and identified all stimuli. Second, the response times were faster for stimuli with form and colour than form only suggesting that less attention was required to name the stimulus when colour was present. In summary, we have located the brain regions where additional perceptual cues (either colour or sound) enhance activation in visual cortices. Although further studies are required to elucidate the precise mechanisms, we propose that the influence of colour and sound occurs prior to the integration of multimodal inputs into a single object representation.

### 3.2 The effect of colour and sound in the right anterior fusiform

In contrast to the posterior occipital regions where activation is associated with early visual processing, the right anterior fusiform has been linked to amodal semantic processing in a number of previous studies. For example, using a repetition priming paradigm, Vuilleumier et al. (2002) showed a repetition decrease in the anterior fusiform (bilaterally) for repetition of real but not nonsense pictures of objects. Martin et al. (1996) reported the same anterior fusiform region (also bilateral) for naming real objects relative to non-objects and Simmons et al. (2007) reported bilateral anterior fusiform activation for retrieving object colour from object names. In the auditory modality, von Kriegstein et al. (2006) demonstrated that familiar voice and face recognition activate the right anterior fusiform, and Thierry and Price (2006) associated the same right anterior fusiform region with amodal conceptual processing of both object sounds and action videos.

The novel finding in the current study is that right anterior fusiform region shows an additive effect when both auditory and visual object inputs are presented simultaneously relative to when auditory and visual inputs are presented independently. There are two possible explanations. One is that increased right fusiform activation reflects the integration of visual and auditory inputs but we think this is unlikely because the advantage of combined inputs was not super-additive relative to form only, sound only or colour only. Furthermore, there was no evidence from the behavioural data that form and sound had been integrated. Indeed, the pattern of response in this fusiform region was strikingly similar to that observed in the bilateral occipital areas, suggesting that activation was driven more by perceptual inputs than by subsequent semantic processes (see Fig. 2). Our preferred explanation, therefore, is that right fusiform activation (like bilateral occipital activation) reflects processing prior to the integration of perceptual cues.

Although category effects have been reported in the fusiform gyri, it is worth noting that the effects observed here do not correspond to regions associated with category selective responses. For example, the right fusiform region activated here is more lateral and anterior to that reported for living > non-living items by Chao et al. (1999). It is not surprising then that no interaction between category and level of perceptual information was observed in this region (see Section 4.5).

### 3.3 The effect of colour and sound in the left antero-medial temporal cortex

The left antero-medial temporal cortex was the only region where we detected reduced activation in the presence of additional perceptual cues, but even here the effect was only identified in the colour-form condition not the sound-form condition. Reduced left antero-medial temporal activation for the addition of appropriate colour cues has previously been reported by Moore and Price (1999) where it was interpreted in terms of a reduction in the number of competing responses within the semantic system. Our finding that response times were facilitated by the addition of colour (see Table 1) is consistent with this explanation. Using the same logic – that faster response times and less activation reflect facilitation – our data also demonstrate that the addition of sound did not facilitate object identification because, when form and sound were combined, there was no difference in response times relative to the form only condition and no decreased left antero-medial temporal activation (see Fig. 3). The most likely explanation is that an object's sound takes longer to process than both form and colour information (see Table 1), therefore there was no advantage of sound when object naming can proceed on the basis of form alone. This does not exclude the possibility that sound would have a more facilitatory influence if the objects were difficult to recognize on the basis of form alone.

The observation that the addition of appropriate colour facilitated picture naming and reduced left medial anterior temporal activation is consistent with Moore and Price (1999). However, we did not observe decreased activation for coloured relative to black and white pictures in the right anterior or posterior temporal regions that were also reported for this contrast in Moore and Price (1999). This inconsistency can be explained by differences in the type of stimuli used. Moore and Price used outline drawings while we used high-resolution photographs. The facilitatory effect of colour on perceptual identification will therefore have been greater in Moore and Price (1999). Indeed, Moore and Price highlight the perceptual role of the right anterior and posterior temporal regions because activation in these areas was higher for drawings of meaningless non-objects than drawings of familiar objects. In contrast, in the left medial anterior temporal region, Moore and Price found greater activation for drawings of objects (particularly fruit) than drawings of meaningless non-objects. Thus, our observation of reduced activation in the left anterior temporal cortex is consistent with the impact of colour on semantic processing but our use of high-resolution photographs may have eliminated the facilitatory effect of colour on perceptual identification.

### 3.4 The absence of an effect of colour and sound in left pSTS

Given the association of pSTS with crossmodal audiovisual processing (for reviews see Calvert, 2001; Calvert and Lewis, 2004; King and Calvert, 2001) it is perhaps surprising that we did not observe increased pSTS activation during the form with sound condition. As discussed above, the most likely explanation is that the object naming task that we used here did not require the integration of form and sound. Rather, naming was possible from the visual input alone, without recourse to the auditory signal. This is consistent with recent evidence demonstrating that superior temporal activation during audiovisual integration is confounded by a range of methodological difficulties (Hocking and Price, 2008).

### 3.5 Summary

To summarize, during a visual object naming task, the addition of colour or sound increased activation in early visual (bilateral occipital) areas as well as areas previously associated with semantic processing (right anterior fusiform). In addition, left antero-medial temporal activation was decreased for combined form/colour inputs but no areas of decreased activation were observed for the combination of visual and sound inputs. This follows the behavioural data where response times were facilitated for the combination of colour and form but not for the combination of sound and form. Increased activation in the occipital and fusiform regions for additional perceptual cues suggests that these effects might be arising prior to the perceptual integration. In contrast, decreased activation in the left antero-medial temporal cortex is likely to occur after form and colour have been integrated.

## 4 Experimental procedures

### 4.1 Subjects

15 subjects (14 males, 1 female, age range 20–65, mean age 32.7) participated in a total of 12 × 90 s PET scans. All were right handed native English speakers with normal or corrected to normal vision. All had normal neurological and audiological status. The study was approved by the joint ethics committee of the Institute of Neurology and University College London Hospital, London, UK. This includes the stipulation that females of child-bearing age cannot be subjected to injection of the radioactive isotope used in PET.

### 4.2 Experimental design and stimuli

We chose to use positron emission tomography (PET) to maximize our chances of detecting anterior temporal lobe activations in areas that are susceptible to inhomogeneities in fMRI and for consistency with the Moore and Price (1999) study. There were 12 PET scans. Three conditions were designed to identify the effect of form (F) and colour (C), with two scans per condition. These were:

(1) Colour photos of objects with a characteristic colour (CF1)
(2) Black and white versions of condition 1 (F1)
(3) Solid colour patches (C1)

The remaining three conditions (two scans per condition) were used to investigate the integration of form (F) and sound (S):

(4) Black and white photos of objects and animals with their characteristic sounds (SF2)
(5) Black and white photographs from condition 4 (F2)
(6) Sounds from condition 4 (S2)

In all conditions, subjects were instructed to articulate their response silently, moving their lips without generating any sounds. The lip movements enabled us to monitor accuracy of response, while the silent responses aimed to minimize auditory processing of the spoken response.

#### 4.2.1 Stimuli

For the conditions investigating the integration of form and colour, stimuli consisted of 48 objects with a prototypical colour (24 manmade and 24 fruits and vegetables) and 12 solid colour patches. The manmade and natural objects were presented in different scans, with 12 items in the CF1 condition and the other 12 in the F1 condition. The same 12 colour patches were repeated twice in the two C1 scans. The form and sound integration conditions consisted of 36 animals and 36 manmade objects all with strongly associated sounds, see Appendix. As in the colour conditions, there were 12 stimuli of the same category per scan, no object was repeated within subject and stimulus set was counterbalanced across subjects.

Photographs were obtained from the Hemara Photo Objects CD collection and object sounds were downloaded from the internet, with the majority obtained from the website www.sounddogs.com. Sounds were converted to mono and were 1500 ms in length. The 12 colour patches were created using Corel Photo-paint v.11, and all visual stimuli were equated as far as possible for size (∼ 8 cm × 8 cm). See Appendix for a complete list and Fig. 1 for examples of each trial type.

To reduce sensory and attention differences between conditions, both visual and auditory stimuli were presented on every trial in every condition. Thus, in the uni-modal auditory condition (S2) each object sound was simultaneously presented with an unrecognizable scrambled photograph (using the “scatter pixel” function in Corel Photo-paint v.11), and in uni-modal visual conditions (CF1, F1, C1, and F2) each visual stimulus was simultaneously presented with an unrecognizable scrambled object sound. Meaningless auditory stimuli were created by converting the object sounds using a Fast Fourier Transform to scramble their frequency. Examples of the stimuli can be seen in Fig. 1.

Stimuli were presented on a 43 cm monitor suspended from a movable gantry at a distance of approximately 50 cm from the subject. Sounds were presented through two speakers situated behind the subject. Stimulus presentation was controlled with COGENT software (www.vislab.ucl.ac.uk). Prior to being scanned, all subjects were familiarized with all stimuli, to ensure that they were equally familiar with the pictures and sounds. Immediately prior to each scan, an instruction was presented on the monitor to indicate what sort of stimuli needed to be named (animal, fruit, manmade object or colour). For each trial, an audiovisual stimulus was presented for 1500 ms, followed by a fixation cross for 2500 ms, giving an inter-stimulus interval of 4000 ms and a total activation block length of 48 s. Although this would be considered to be long for an fMRI study, 48 s is considered optimum for PET (Silbersweig et al., 1993).

### 4.3 Behavioural study

As it was only possible to record accuracy but not response latencies in the scanner, an additional behavioural study was run with a separate group of 24 subjects (age range: 24–55, mean age 32.2) to investigate differences in naming latencies for each condition. These subjects received the same instructions and the same 12 conditions as the subjects in the PET study, and were instructed to name the stimuli out loud as quickly and as accurately as possible. Stimulus presentation was delivered on a laptop computer, using the same presentation software as during scanning, in a private testing room, with only the subject and an experimenter present. Response latencies were recorded with a voice activated relay system from stimulus to response onset.

### 4.4 Data acquisition

All subjects underwent 12 PET relative perfusion scans at the Wellcome Trust Centre for Neuroimaging, London, UK. Scans were obtained using a Siemens/CPS ECAT Exact HR+ (model 962) head scanner (Siemens/CTI, Knoxville, TN, USA) with a total field view of 15 cm. The head of each subject was located in the centre of the PET camera by means of a helmet attached with Velcro to the scanner bed in order to minimize movement within and between each scan. They received a 20 s intravenous bolus of H_2_^15^O at a concentration of 55 MBq/ml and a flow rate of 10 ml/min through a forearm venous cannula. For each scan, approximately 10–15 mCi of H_2_^15^O in 3 m of normal saline was flushed into the subject over 20 s, at a rate of 10 ml/min by an automatic pump. After a 30 s background scan, head counts peaked 30–40 s later (depending on the individuals' circulation time). Data acquisition time lasted 90 s, with an interval of 9 min between successive H_2_^15^O administrations. The assimilated radioactivity counts accumulated over the 90 s acquisition period were corrected for background noise and were used as an index of regional cerebral blood flow (rCBF).

Attenuation was corrected for by performing a transmission scan at the beginning of each study with an exposed ^68^Ge/^68^Ga external source. Images were reconstructed in 3D filtered back projection (Hanning filter, cut off frequency 0.5 Hz), giving a transaxial resolution of 8.5 mm full width at half maximum. The reconstructed images contained 128 × 128 pixels, each 2.05 × 2.05 × 2.00 mm in size. To ensure normal neurological status a T1-weighted structural MRI was also obtained for each participant with a Siemens Magnetom Vision 1.5T scanner (Siemens, Erlangen, Germany).

### 4.5 Data transformation

After realignment and spatial normalization of each scan to a reference PET template (Friston et al., 1995a) that conformed to the standard MNI space, all images were smoothed with a Gaussian kernel of 10 mm FWHM. Statistical analysis involved Analysis of Covariance (ANCOVA) with subject effects modelled and global activity included as a subject specific covariate. The condition and subject effects were estimated according to the general linear model at each voxel (Friston et al., 1995b). The resulting set of voxel values constitutes a SPM of the *t* statistic (SPMt), the values of which were transformed to the unit normal distribution (SPMZ).

In a preliminary analysis that modelled living and non-living object categories separately, the effects of additional perceptual information did not interact with category. The results reported in this paper are therefore based on an analysis that summed over the effect of object category. Nevertheless, we note here that in addition to the results reported in this paper, we also observed a highly significant effect of object category in the left posterior middle temporal cortex. As reported in many previous studies (e.g. Devlin et al., 2002; Lewis et al., 2005), left posterior middle temporal activation was higher when the stimuli were manmade objects than living entities (animals, fruits and vegetables).

In the analysis of 6 conditions, we identified where activation was increased or decreased with the addition of colour and sound using the following contrasts:

(1) Positive main effect of additional perceptual cues = [CF1+SF2 > C1+F1+S2+F2] and negative main effect of additional perceptual cues = [CF1+SF2 < C1+F1+S2+F2]
(2) Interaction of additional perceptual cues with sensory modality:
  a. Increased for addition of colour/decreased for addition of sound = [CF1 > C1 + F1] − [SF2 > S2 + F2]
  b. Increased for addition of sound or decreased for addition of colour = [SF2 > S2 + F2] − [CF1 > C1 + F1]
(3) Simple main effects of:
  a. increased for additional colour = [CF1 > C1 + F1]
  b. decreased additional colour = [CF1 < C1 + F1]
  c. increased for additional sound = [SF2 > S2 + F2]
  d. decreased for additional sound = [SF2 < S2 + F2]

We report only those effects that reach a threshold of *p* < 0.05, family wise error corrected for multiple comparisons either across the whole brain or in two regions of interest (ROIs) based on previous literature. The first ROI was taken from the Moore and Price (1999) study, where facilitation was observed for coloured relative to black and white natural objects in the antero-medial temporal cortices at the co-ordinates [− 26, 0,− 20] and [30, 8,− 24] and the right posterior temporal cortex centred around [64,− 56, 0]. The second ROI was the pSTS region associated with enhanced activation during audiovisual integration. It was centred on the peak co-ordinates reported in Beauchamp et al. (2004) at [− 50,− 55, 7] for nonverbal audiovisual integration. These co-ordinates were converted from Talairach and Tournoux stereotactic space into the nearest estimated co-ordinates in MNI space [+/− 50,− 56, 4] using the algorithm developed by Matthew Brett (http://www.mrc-cbu.cam.ac.uk/Imaging). Within our two ROIs, the search volume was a sphere of 10 mm radius.

## Figures and Tables

**Table:** 

*Manmade object with a prototypical colour*					
Ambulance	Battery	Fire engine	Lego	Ring	Tennis ball
Baseball bat	Brick	Fire extinguisher	Lipstick	Rolling pin	Traffic cone
Basket	Coin	Flag	Postbox	Taxi	Traffic light
Basketball	London bus	Land rover	Red wine bottle	Telephone box	Wooden spoon
*Natural objects with a prototypical colour*					
Apple	Carrot	Garlic	Melon	Peach	Potato
Banana	Coconut	Leek	Mushroom	Pear	Raspberry
Bean	Corn	Lemon	Onion	Pepper	Strawberry
Broccoli	Courgette	Lettuce	Orange	Pineapple	Tomato
*Manmade objects with a prototypical object sound*					
Accordion	Clarinet	Door	Guitar	Phone	Toilet
Bell	Clock	Doorbell	Gun	Piano	Toothbrush
Bicycle bell	Coin	Drum	Hammer	Plane	Train
Bongos'	Cork	Fire engine	Harp	Razor	Trumpet
Camera	Cymbals	Flute	Lawnmower	Saw	Whistle
Can	Dice	Gong	Match	Tap	Zip
*Natural objects with a prototypical sound*					
Baby (crying)	Cow	Donkey	Goose	Parrot	Sheep
Baby (laughing)	Cricket	Duck	Horse	Pig	Snake
Bee	Crow	Elephant	Horse	Rattlesnake	Sparrow
Cat	Cuckoo	Fly	Lion	Rooster	Whale
Cat	Dog (barking)	Frog	Mosquito	Seagull	Wolf
Chicken	Dog (yapping)	Goat	Owl	Seal	Wood pigeon
*Colours*					
Black	Brown	Green	Orange	Purple	White
Blue	Cream	Grey	Pink	Red	Yellow

**Table 1:** Naming latencies (mean and standard deviation) for behavioural experiment

Naming condition	Mean (ms)	SD (ms)
CF1	932	99
F1	966	105
C1	799	114
SF2	960	130
F2	961	162
S2	1556	273

Key: C = colour; F = form; S = sound; 1 = vision only; 2 = audiovisual.

**Table 2:** Regional activation for main effect of increased perceptual input

Main effects				*Z*-scores for individual contracts			
Anatomical region	Peak cluster	*Z*-score (CF1>C1+F1) +(SF2>S2+F2)	Number of voxels	CF1>C1	CF1>F1	SF2>S2	SF2>F2
R inf/mid occipital	**34,** − **98,** − **4**	6.1	740	5.4	2.9	5.3	1.7
	*30,* − *90,* − *10*	4.8		4.0	1.6	5.1	2.8
L inf/mid occipital	− **32,** − **92,** − **18**	5.1	581	5.5	2.7	3.9	1.8
R ant fusiform	**42,** − **40,** − **14**	4.2	239	2.8	1.8	3.8	2.3
	*36* − *34* − *12*	3.2		2.2	3.5	1.6	1.6

Table 2, columns 1–4, shows the anatomical regions, MNI co-ordinates (centre of peak cluster shown in bold) and corresponding *Z*-scores for the main effect of increased perceptual cues across input type. Columns 5–8 show the *Z*-scores for the individual linear contrasts.

## References

[bib1] Barbarotto R., Capitani E., Laiacona M. (1996). Naming deficit in herpes simplex encephalitis. Acta Neurol. Scand..

[bib2] Bartels A., Zeki S. (2000). The architecture of the colour centre in the human visual brain: new results and a review. Eur. J. Neurosci..

[bib3] Bartels A., Zeki S. (2006). The temporal order of binding visual attributes. Vis. Res..

[bib4] Beauchamp M.S., Lee K.E., Argall B.D., Martin A. (2004). Integration of auditory and visual information about objects in superior temporal sulcus. Neuron.

[bib5] Bookheimer S.Y., Zeffiro T.A., Blaxton T.A., Gaillard W.D., Theodore W.H. (1995). Regional cerebral blood flow during object naming and word reading. Hum. Brain. Mapp*.*.

[bib6] Bookheimer S.Y., Zeffiro T.A., Blaxton T.A., Gaillard W.D., Malow B., Theodore W.H. (1998). Regional cerebral blood flow during auditory responsive naming: evidence for cross-modality neural activation. Neuroreport.

[bib7] Booth J.R., Burman D.D., Meyer J.R., Gitelman D.R., Parrish T.B., Mesulam M.M. (2002). Modality independence of word comprehension. Hum. Brain. Mapp..

[bib8] Brambati S.M., Myers D., Wilson A., Rankin K.P., Allison S.C., Rosen H.J., Miller B.L., Gorno-Tempini M.L. (2006). The anatomy of category-specific object naming in neurodegenerative diseases. J. Cogn. Neurosci..

[bib9] Calvert G.A. (2001). Crossmodal processing in the human brain: insights from functional neuroimaging studies. Cereb. Cortex.

[bib10] Calvert G.A., Lewis J.W., Calvert G.A., Spence C., Stein B.E. (2004). Hemodynamic studies of audiovisual interactions. The Handbook of Multisensory Processes.

[bib11] Cant J.S., Goodale M.A. (2007). Attention to form or surface properties modulates different regions of human occipitotemporal cortex. Cereb. Cortex.

[bib12] Chao L.L., Haxby J.V., Martin A. (1999). Attribute-based neural substrates in temporal cortex for perceiving and knowing about objects. Nat. Neurosci..

[bib13] Clarke S., Bellmann A., Meuli R.A., Assal G., Steck A.J. (2000). Auditory agnosia and auditory spatial deficits following left hemispheric lesions: evidence for distinct processing pathways. Neuropsychologia.

[bib14] Davies R.R., Graham K.S., Xuereb J.H., Williams G.B., Hodges J.R. (2004). The human perirhinal cortex and semantic memory. Eur. J. Neurosci..

[bib15] Devlin J.T., Moore C.J., Mummery C.J., Gorno-Tempini M.L., Phillips J.A., Noppeney U., Frackowiak R.S., Friston K.J., Price C.J. (2002). Anatomic constraints on cognitive theories of category specificity. Neuroimage.

[bib16] Dojat M., Piettre L., Delon-Martin C., Pachot-Clouard M., Segebarth C., Knoblauch K. (2006). Global integration of local color differences in transparency perception: an fMRI study. Vis. Neurosci..

[bib17] Friston K.J., Ashburner J., Frith C.D., Poline J.B., Heather J.D., Frackowiak R.S.J. (1995). Spatial registration and normalization of images. Hum. Brain Mapp..

[bib18] Friston K.J., Holmes A., Worsley K., Poline J.B., Frith C.D., Frackowiak R.S.J. (1995). Statistical parametric maps in functional imaging: a general linear approach. Hum. Brain Mapp..

[bib19] Giard M.H., Peronnet F. (1999). Auditory-visual integration during multimodal object recognition in humans: a behavioral and electrophysiological study. J. Cogn. Neurosci..

[bib20] Grill-Spector K. (2003). The neural basis of object perception. Curr. Opin. Neurobiol..

[bib21] Hadjikhani N., Liu A.K., Dale A.M., Cavanagh P., Tootell R.B. (1998). Retinotopy and color sensitivity in human visual cortical area V8. Nat. Neurosci..

[bib22] Hein G., Doehrmann O., Muller N.G., Kaiser J., Muckli L., Naumer M.J. (2007). Object familiarity and semantic congruency modulate responses in cortical audiovisual integration areas. J. Neurosci..

[bib23] Hershenson M. (1962). Reaction time as a measure of intersensory facilitation. J. Exp. Psychol..

[bib24] Hocking J., Price C.J. (2008). The role of the posterior superior temporal sulcus in audiovisual processing. Cereb Cortex.

[bib25] Howard R.J., Ffytche D.H., Barnes J., McKeefry D., Ha Y., Woodruff P.W., Bullmore E.T., Simmons A., Williams S.C., David A.S., Brammer M. (1998). The functional anatomy of imagining and perceiving colour. Neuroreport.

[bib26] Humphrey G.K., Goodale M.A., Jakobson L.S., Servos P. (1994). The role of surface information in object recognition: studies of a visual form agnosic and normal subjects. Perception.

[bib27] Humphreys G.W., Lamote C., Lloyd-Jones T.J. (1995). An interactive activation approach to object processing: effects of structural similarity, name frequency, and task in normality and pathology. Memory.

[bib28] Joseph J.E., Proffitt D.R. (1996). Semantic versus perceptual influences of color in object recognition. J. Exp. Psychol. Learn. Mem. Cogn..

[bib29] Joseph J.E., Gathers A.D. (2003). Effects of structural similarity on neural substrates for object recognition. Cogn. Affect. Behav. Neurosci..

[bib30] Kapur N., Ellison D., Parkin A.J., Hunkin N.M., Burrows E., Sampson S.A., Morrison E.A. (1994). Bilateral temporal lobe pathology with sparing of medial temporal lobe structures: lesion profile and pattern of memory disorder. Neuropsychologia.

[bib31] King A.J., Calvert G.A. (2001). Multisensory integration: perceptual grouping by eye and ear. Curr. Biol..

[bib32] Laurienti P.J., Wallace M.T., Maldjian J.A., Susi C.M., Stein B.E., Burdette J.H. (2003). Cross-modal sensory processing in the anterior cingulate and medial prefrontal cortices. Hum. Brain Mapp..

[bib33] Lewis J.W., Brefczynski J.A., Phinney R.E., Janik J.J., DeYoe E.A. (2005). Distinct cortical pathways for processing tool versus animal sounds. J. Neurosci..

[bib34] Maeder P.P., Meuli R.A., Adriani M., Bellmann A., Fornari E., Thiran J.P., Pittet A., Clarke S. (2001). Distinct pathways involved in sound recognition and localization: a human fMRI study. Neuroimage.

[bib35] Mapelli D., Behrmann M. (1997). The role of color in object recognition: evidence from visual agnosia. Neurocase.

[bib36] Martin A. (2007). The representation of object concepts in the brain. Annu. Rev. Psychol..

[bib37] Martin A., Wiggs C.L., Ungerleider L.G., Haxby J.V. (1996). Neural correlates of category-specific knowledge. Nature.

[bib38] McKeefry D.J., Zeki S. (1997). The position and topography of the human colour centre as revealed by functional magnetic resonance imaging. Brain.

[bib39] McRae K., Cree G.S., Westmacott R., de Sa V.R. (1999). Further evidence for feature correlations in semantic memory. Can. J. Exp. Psychol..

[bib40] Moore C.J., Price C.J. (1999). A functional neuroimaging study of the variables that generate category-specific object processing differences. Brain.

[bib41] Noppeney U., Patterson K., Tyler L.K., Moss H., Stamatakis E.A., Bright P., Mummery C., Price C.J. (2007). Temporal lobe lesions and semantic impairment: a comparison of herpes simplex virus encephalitis and semantic dementia. Brain.

[bib42] Nunn J.A., Gregory L.J., Brammer M., Williams S.C., Parslow D.M., Morgan M.J., Morris R.G., Bullmore E.T., Baron-Cohen S., Gray J.A. (2002). Functional magnetic resonance imaging of synesthesia: activation of V4/V8 by spoken words. Nat. Neurosci..

[bib43] Nyberg L., Habib R., McIntosh A.R., Tulving E. (2000). Reactivation of encoding-related brain activity during memory retrieval. Proc. Natl. Acad. Sci. U. S. A..

[bib44] Ostergaard A.L., Davidoff J.B. (1985). Some effects of color on naming and recognition of objects. J. Exp. Psychol. Learn. Mem. Cogn..

[bib45] Price C.J., Humphreys G.W. (1989). The effects of surface detail on object categorization and naming. Q. J. Exp. Psychol. A.

[bib46] Rauschecker J.P., Tian B. (2000). Mechanisms and streams for processing of “what” and “where” in auditory cortex. Proc. Natl. Acad. Sci. U. S. A..

[bib47] Sakai K., Watanabe E., Onodera Y., Uchida I., Kato H., Yamamoto E., Koizumi H., Miyashita Y. (1995). Functional mapping of the human colour centre with echo-planar magnetic resonance imaging. Proc. Biol. Sci..

[bib48] Silbersweig D.A., Stern E., Frith C.D., Cahill C., Schnorr L., Grootoonk S., Spinks T., Clark J., Frackowiak R., Jones T. (1993). Detection of thirty-second cognitive activations in single subjects with positron emission tomography: a new low-dose H2(15)O regional cerebral blood flow three-dimensional imaging technique. J. Cereb. Blood Flow Metab..

[bib49] Simmons W.K., Ramjee V., Beauchamp M.S., McRae K., Martin A., Barsalou L.W. (2007). A common neural substrate for perceiving and knowing about color. Neuropsychologia.

[bib50] Spitsyna G., Warren J.E., Scott S.K., Turkheimer F.E., Wise R.J. (2006). Converging language streams in the human temporal lobe. J. Neurosci..

[bib51] Summerfield Q. (1992). Lipreading and audio-visual speech perception. Philos. Trans. R. Soc. Lond. B Biol. Sci..

[bib52] Taylor K.I., Moss H.E., Stamatakis E.A., Tyler L.K. (2006). Binding crossmodal object features in perirhinal cortex. Proc. Natl. Acad. Sci. U.S.A.

[bib53] Teder-Sälejärvi W.A., Di Russo F., McDonald J.J., Hillyard S.A. (2005). Effects of spatial congruity on audio-visual multimodal integration. J. Cogn. Neurosci..

[bib54] Thierry G., Price C.J. (2006). Dissociating verbal and nonverbal conceptual processing in the human brain. J. Cogn. Neurosci..

[bib55] von Kriegstein K., Kleinschmidt A., Giraud A.L. (2006). Voice recognition and cross-modal responses to familiar speakers' voices in prosopagnosia. Cereb. Cortex.

[bib56] Vuilleumier P., Henson R.N., Driver J., Dolan R.J. (2002). Multiple levels of visual object constancy revealed by event-related fMRI of repetition priming. Nat. Neurosci..

[bib57] Wheeler M.E., Petersen S.E., Buckner R.L. (2000). Memory's echo: vivid remembering reactivates sensory-specific cortex. Proc. Natl. Acad. Sci. U. S. A..

[bib58] Zeki S., Marini L. (1998). Three cortical stages of colour processing in the human brain. Brain.

[bib59] Zeki S., Watson J.D., Lueck C.J., Friston K.J., Kennard C., Frackowiak R.S. (1991). A direct demonstration of functional specialization in human visual cortex. J. Neurosci..

